# Systematic Assessment of Flavor Cues and Additives in Cigarettes and Heated Tobacco Products in Korea: Cross-Sectional Surveillance Study

**DOI:** 10.2196/87537

**Published:** 2026-05-25

**Authors:** Geon Heo, Doyoon Kim, Yehyun Kim, Ara Kim, Nagyeong Cho, Naeun Kang, Jungmi Park, Eunsil Cheon, Seulgi Kim, Susan Park, Sungmin Park, Sung-il Cho, Heewon Kang

**Affiliations:** 1 Department of Public Health Sciences, Graduate School of Public Health, Seoul National University, Seoul, Republic of Korea; 2 Armed Forces Capital Hospital, Seongnam, Republic of Korea; 3 Institute of Health and Environment, Seoul National University, Seoul, Republic of Korea; 4 School of Medicine, Inha University, Incheon, Republic of Korea; 5 Department of Health Administration, Daejin University, 1007 Hoguk-ro, Pocheon-si, Gyeonggi-do, Gyeonggi, 11159, Republic of Korea, 82 31 539 1884

**Keywords:** cigarettes, heated tobacco products, packaging, flavor cues, flavor additives, regulation

## Abstract

**Background:**

In South Korea, where plain packaging has not been adopted, tobacco packaging continues to function as a key marketing tool for the tobacco industry, using texts, colors, and imagery to attract consumers. Among these, flavor cues are especially important as they enhance product appeal. Cigarette sticks also serve marketing functions through design features such as colors and capsule indicators.

**Objective:**

This study aimed to examine flavor-related cues on cigarettes and heated tobacco products (HTPs) packaging and stick design and to assess the presence of flavor additives in these products.

**Methods:**

This surveillance study was conducted in November 2024. Tobacco products were purchased from convenience stores located in Seoul, supplemented by cross-referencing with national market monitoring data. Of 353 identified products, 214 products (150 cigarettes and 64 HTPs) were collected. Flavor cues were categorized by pack and stick design features, and additives were identified through sensory analysis of product components.

**Results:**

Among the collected products, 63.1% (54% for cigarettes and 84.4% for HTPs) had both flavor cues and flavor additives, while 20.6% (27.3% for cigarettes and 4.7% for HTPs) had neither. Flavor cues were found in 67.3% of cigarettes and 95.3% of HTPs (*P*<.001), and flavor additives in 59.3% of cigarettes and 84.4% of HTPs (*P*<.001). Pack color was the most common cue, and additives were most often delivered through crushable capsules. HTPs used a wider range of flavoring methods, including flavoring in tobacco leaves and inner wrappers.

**Conclusions:**

Tobacco packaging and stick design in South Korea remain important marketing tools for the tobacco industry. Flavor cues and additives are widely used in tobacco products, particularly in HTPs. These findings highlight the need for plain packaging policies and bans on flavor additives in tobacco products.

## Introduction

Tobacco packaging is the first element of the product that people encounter, and it functions as a key marketing tool for the tobacco industry [1], particularly in settings where plain packaging has not been implemented [2]. Packaging elements such as color, imagery, and text can evoke positive perceptions and stimulate tobacco use, particularly among youth and individuals who have quit smoking [3-6]. Accordingly, Article 11 of the Framework Convention on Tobacco Control (FCTC) emphasizes strict regulation of promotional features on tobacco packs and labels [7], and several countries have adopted plain packaging and enlarged health warnings to reduce product appeal [8]. These measures have also been extended to cigarette sticks, restricting the use of stick appearance as a marketing tool [9]. Experimental evidence shows that unattractive stick colors or warning messages on sticks significantly reduce appeal and purchase intentions, suggesting that plain packaging should extend beyond packs to include sticks [9,10].

Flavor-related experiences are frequently communicated through tobacco packaging using contextual and visual cues [11-15]. In settings without plain packaging, manufacturers use concept descriptors (eg, “Ice” and “Blue”) in combination with color schemes to signal taste and sensation without explicitly naming a characterizing flavor [12,16]. These cues shape consumer perceptions, and products with flavor cues are often perceived as more appealing, better tasting, and smoother than nonflavored or standardized variants [13]. This sensation transfer reduces perceived harshness and creates a health halo that leads consumers, especially youth and young adults, to perceive these products as less harmful [11,14].

To effectively implement and evaluate packaging regulations, systematic surveillance of tobacco packs is crucial. Much of the evidence base for assessing tobacco packaging has been shaped by the Tobacco Pack Surveillance System (TPackSS), developed by the Institute for Global Tobacco Control [11,12,17]. These studies demonstrate how systematic observation can identify flavor cues and other tactics used to enhance product appeal, providing evidence to support stronger regulations [11,12,17]. Recent pack surveillance in the Philippines showed that nearly 60% of tobacco products had explicit flavor cues, often reinforced by imagery, highlighting the persistent appeal of flavored products in the absence of regulation [11].

Building on this evidence, systematic surveillance is particularly important for addressing flavored tobacco products. This aligns with the recommendations of Articles 9 and 10 of the FCTC, which emphasize the need to monitor and regulate product contents, including flavoring [18]. Flavored tobacco products mask the harshness of nicotine and attract youth, women, and racial and ethnic minority groups [19-22]. They make tobacco use more appealing, increase nicotine dependence, and undermine quitting [21,23]. In response to this evidence, an increasing number of countries have banned flavored cigarettes [24]. However, most surveillance studies have primarily focused on cigarettes, with limited assessment of flavor cues in heated tobacco products (HTPs).

In South Korea (hereafter referred to as Korea), this issue has become particularly pressing. Following 3 decades of decline, smoking prevalence rebounded to 19.6% among adults in 2023. Although it declined to 16.7% in 2024, it remains high (28.5% among men and 4.2% among women) [25]. This trend has coincided with increased sales of flavored tobacco products, driven predominantly by flavor capsule cigarettes and HTPs [26]. In 2023, 1.68 billion packs of flavored cigarettes and HTPs were sold, accounting for 46.5% of total tobacco sales (3.61 billion packs). This marks an increase from 270 million packs (6.1%) in 2011 and 860 million packs (24.4%) in 2017 [26].

Despite the rapidly increasing sales of flavored cigarettes and HTPs, Korea still lacks comprehensive regulatory measures addressing flavor-related marketing. Although flavored tobacco products remain legal to manufacture and sell, Article 9‐3 of the National Health Promotion Act (NHPA) prohibits the use of explicit text or imagery indicating flavors on tobacco packaging [27]. However, enforcement has been limited. Indirect flavor cues are also not regulated, allowing manufacturers to imply flavors through concept descriptors, colors, and other design elements [27].

In addition, while the NHPA mandates health warnings covering at least 50% of the main surfaces, with pictorial warnings accounting for at least 30% [27], Korea has not adopted plain packaging, despite its inclusion in the 2019 Tobacco Control Plan [28]. Other measures outlined in the Plan, such as a ban on flavored additives, also remain unimplemented. Moreover, there is no display ban at retail points of sale, and tobacco packs are predominantly showcased on power walls [27]. Together, these gaps leave visitors to approximately 140,000 tobacco retailers (primarily convenience stores and supermarkets) [29] continuously exposed to tobacco marketing, thereby increasing product appeal and the likelihood of tobacco use, particularly among youth [1,4,5].

In this study, we conducted a systematic assessment of cigarettes and HTPs sold in Korea, examining pack- and stick-level flavor cues as well as flavor additives at the component level. By combining pack- and stick-level surveillance with component-based sensory screening, we address a key gap in the literature on how flavor marketing cues align with flavoring and how these patterns differ between cigarettes and HTPs. We aimed to generate evidence to inform regulation and strengthen surveillance in Korea and internationally.

## Methods

### Study Design

To assess the packaging of cigarettes and HTPs sold in Korea, we used a market-based purposive sampling strategy designed to capture the diversity of unique brands and pack variants available on the market, rather than to estimate sales volume or market share [17]. Our pack documentation and photography procedures were informed by the TPackSS protocol [17], and we coded pack-level flavor cues such as descriptors, imagery, and capsules based on prior pack surveillance research [11,12]. Furthermore, drawing on prior studies [30-32], we developed an exploratory protocol to cut open individual cigarettes and HTP sticks (hereafter referred to as sticks) to examine internal flavor-related features. We also documented external flavor cues, including capsule indicators on sticks, before disassembly.

### Sample

**Figure 1.**
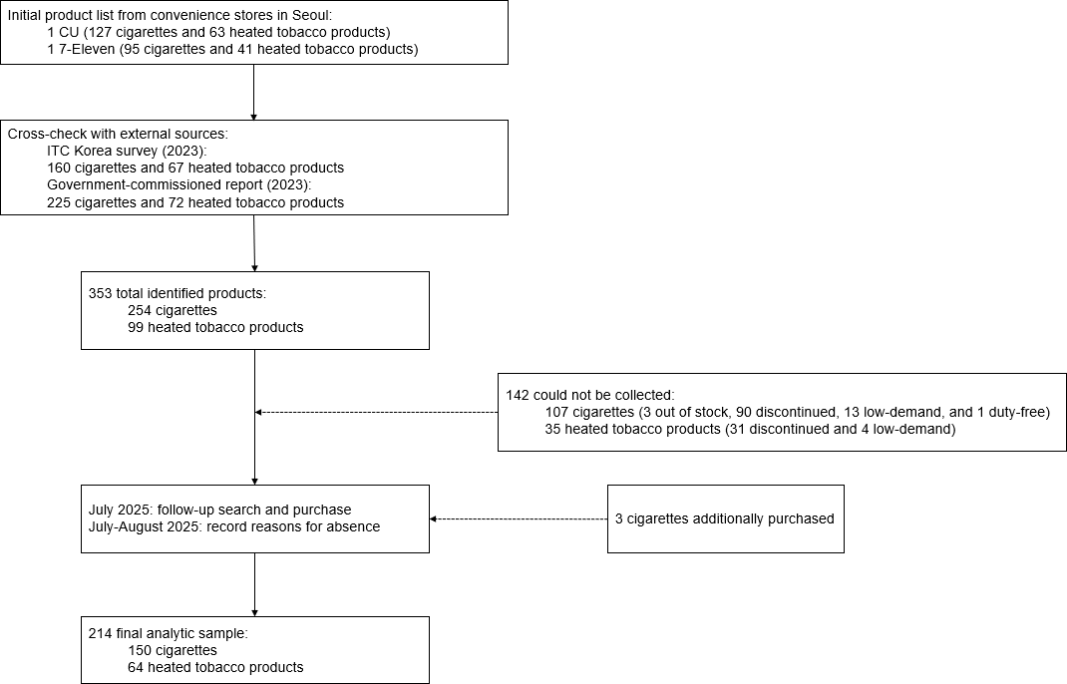
Product identification and inclusion flow.

The product identification process involved four steps: (1) compiling an initial product list, (2) purchasing available products, (3) cross-referencing with external data sources, and (4) investigating reasons for unavailable products (Figure 1). To construct an initial product list, we visited 1 retail store from each of the 2 major convenience store chains in Seoul (CU and 7-Eleven) in November 2024 to collect all available tobacco products. We focused exclusively on convenience stores because they represent the predominant retail channel for tobacco sales in South Korea [29]. Among these, CU and 7-Eleven were selected due to their market dominance; together, they account for 56.3% of all convenience stores nationwide [33]. Within each chain, 1 store in Seoul was selected based on geographic proximity to the research team. Observational checks at additional outlets suggested that product availability was comparable across stores.

All tobacco products available in the visited stores were purchased, including all unique retail pack variants available at the point of sale. The initial list of purchased products was cross-checked against 2 external sources: the Wave 3 (2023) International Tobacco Control Korea Survey [34], which listed 160 cigarettes and 67 HTP brands, and a government-commissioned tobacco product market report [35], which identified 225 cigarettes and 72 HTP products purchased from 46 convenience stores nationwide in October 2023.

Products missing from the initial list (142 products: 107 cigarettes and 35 HTPs) were assessed using external sources to determine their market status. Products were classified as discontinued or low-demand based on Euromonitor reports (market share <0.3%) [26] and corroborating evidence from news articles and websites describing products as “discontinued,” “low-demand,” or “unpopular.” Duty-free exclusive products were verified through media reports. Among missing cigarettes, 90 (84.1%) were discontinued, 13 (12.1%) were not stocked due to low-demand, 3 (2.8%) were out of stock at the time of purchase, and 1 (0.9%) was duty-free exclusive and therefore excluded. Among missing HTPs, 31 (88.6%) were discontinued, and 4 (11.4%) were not stocked due to low demand.

Products not classified as discontinued, low-demand, or duty-free exclusives were subsequently sought through additional visits in July 2025, resulting in the collection of 3 additional packs. In total, 254 cigarettes and 99 HTPs were identified across all sources, of which 104 (40.9%) cigarettes and 35 (35.3%) HTPs were not collected. Reasons for noncollection are documented in Tables S1 and S2 in Multimedia Appendix 1.

### Coding Procedure and Measures

#### Overview

After collection, we extracted and coded relevant information based on the assessment guidelines. HK developed a draft codebook, informed by previous pack- and stick-level assessment frameworks, to capture flavoring components in cigarettes and HTPs [11,12,17,32]. The codebook was reviewed and refined by SK and Susan Park. The final codebook included predefined variables with fixed response options and illustrative examples (where applicable).

Three coding teams (team 1: GH, YK, and NC; team 2: DK, AK, and JP; and team 3: EC and NK) assessed the products, with each product coded by 1 team. Each team consisted of 2 or 3 coders who jointly reviewed each product and recorded a single consensus code. Unresolved cases were cross-checked by another team. For data quality control, the final dataset was checked for internal consistency (logical validity across variables) and missingness. As coding was consensus-based and only final codes were retained, intercoder reliability statistics were not calculated. All coding was conducted collaboratively using a shared online spreadsheet.

#### Visual Measures

Visual measures captured visible pack [11,12,36] and stick features. Packs were measured for size and photographed before and after opening using the TPackSS photography protocol [17], and sticks were measured and photographed using a study-developed protocol (Figures S1 and S2 in Multimedia Appendix 1). Coding was conducted using photographs alongside direct inspection of the physical packs [17] and sticks.

We recorded whether tar and nicotine yields were displayed on the exterior of the cigarette package. Compliance with mandated health warnings was assessed by checking their presence and placement in accordance with national regulatory requirements [37]. Pack shape was coded based on the overall external form of the package. Marketing text was coded as text promoting product features or technological attributes, excluding legally required warnings.

Pack-level flavor cues were identified across 5 elements (Figure S3 in Multimedia Appendix 1): product name, pack color, capsule imagery, pack imagery, and other features [11]. Stick-level flavor cues were additionally coded based on the presence of capsule imagery. Coders were not blinded to brand names or pack descriptors and marketing language, because the study aimed to document consumer-facing packaging cues.

#### Flavor Cue Coding

Flavor cues were coded using a 2-step approach. First, coders recorded the presence or absence of a cue across the 5 pack elements. Second, for cued elements, coders assigned a predefined descriptive category (eg, product name: “cool or minty,” “color,” “flavor,” “functional,” and “others”) to document how the cue was communicated. Thus, the primary quantitative measure was the binary presence or absence of each cue element, while descriptive labels were used to characterize how cues were expressed across products.

Product names were coded as flavor cues when they contained semantic or lexical descriptors implying a specific flavor(“eg, “Raison Ice Presso”), color-associated sensation (eg, “Mevius Mix Green”), or sensory experience (eg, coolness or sweetness), rather than functioning solely as brand or variant names (eg, “Esse Prime”). This distinction clarified that “cool or minty” terms represented perceived freshness rather than literal colors (eg, aqua, cool shot, and ice). Within the product name category, “cool or minty” cues were treated as a distinct subgroup given the particularly prominent impact on perceptions of reduced sensory harshness and greater appeal [38].

Pack color and capsule imagery were coded by dominant color tones on the principal display panel and grouped into 4 sensory color groups: cool or minty, fruity, nutty, and others [11,12,16,39]. Previous experimental research has shown that the appeal and interpretation of pack color and capsule imagery may vary across flavor contexts, including fruity, nutty, and cool or minty categories, supporting the use of descriptive sensory groupings in packaging research [4,40]. Dominant color was defined as the most visually prominent color on the principal display panel, and only colors corresponding to flavor-related conventions reported in prior tobacco packaging research [4,16,41] were coded as flavor cues. These groupings descriptively captured packaging conventions in which color signals expected sensations [4]. More broadly, evidence on crossmodal correspondences shows that package color can shape flavor and sensory perceptions even without explicit flavor descriptors [42].

Additionally, we coded animal imagery as implicit flavor cues. Animal imagery (eg, penguins and polar bears) was included because such visuals function as established metaphors for coolness or freshness in tobacco marketing [43]. The “Others” category included textual statements promoting a cool or minty sensation (eg, Cuban coolness inside) or presence of flavor (eg, max flavor), which were evaluated as presented on the packs without interpretive modification. The complete coding framework with detailed criteria is presented in Table 1 and Figure S4 in Multimedia Appendix 1.

**Table 1.** Tobacco product and packaging characteristics codebook.

Main category and subcategory	Class	Evaluation criteria and examples	Categorization^a^
Basic characteristics			
Product type	Cigarette Heated tobacco product (HTP)	ESSE, MARLBORO, DUNHILL, MEVIUS, etc. TEREA, FiiT, NEO, etc.	—^b^
Manufacturers	BAT JTI PMIK KT&G	DUNHILL, ROTHMAN, glo, etc. MEVIUS, CAMEL, etc. MARLBORO, PARLIAMENT, TEREA, etc. ESSE, RAISON, FiiT, MIIX, etc.	—
Price	Continuous data	Fill in the price written on the receipt.	<4500 KRW^c^ =4500 KRW >4500 KRW
Number of sticks per pack	14 20	Fill in the form after opening the pack.	—
Packaging characteristic			
Tar content (mg)^d^	Continuous data	Fill in the form referring to the left or right side of the tobacco pack; not applicable for HTPs.	<2.7 mg ≥2.7 mg
Nicotine content (mg)^d^	Continuous data	Fill in the form referring to the left or right side of the tobacco pack; not applicable for HTPs.	<0.2 mg =0.2 mg >0.2 mg
Cigarette stick length (mm)	Continuous data	Fill in the form after measuring the length of the cigarette stick.	<79 mm ≥79 mm
Health warning compliance	Yes No	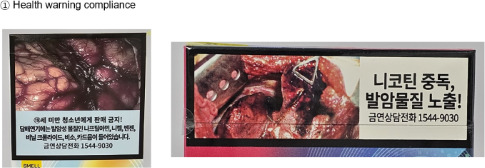	—
Marketing text	Yes No	“Smell Care,” “SENSIBLE SLIM,” “Innovation SUPER CARBON FILTER,” survey results, etc.	—
Dimension characteristic			
Pack depth (mm)	Continuous data	Fill in the form after measuring the length of the applicable part.	<60 mm ≥60 mm
Pack width (mm)	Continuous data	Fill in the form after measuring the length of the applicable part.	<17 mm ≥17 mm
Pack height (mm)	Continuous data	Fill in the form after measuring the length of the applicable part.	<82 mm ≥82 mm
Pack shape	Rectangle Round-edged rectangle Half-round-edged rectangle	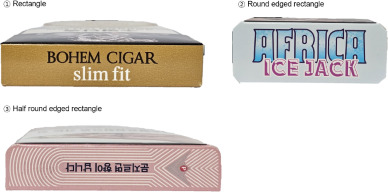	—
Flavor cue^e^ [11,12,16,38]			
Product name	Flavor cued Flavor not cued	If flavor cued, classified into the following subtypes: Cool or minty (eg, frozen and ice) Color (eg, green and blue) Flavor (eg, tropical and pearl), Functional (eg, boost and hybrid) Other (eg, beach and blossom)	—
Pack color	Flavor cued Flavor not cued	If flavor cued, classified into the following subtypes: Cool or minty (eg, blue and green) Fruity (eg, orange and pink) Nutty (eg, brown and yellow) Other (eg, purple)	—
Capsule imagery	Flavor cued Flavor not cued	If flavor cued, classified into the following subtypes: Cool or minty (eg, blue and green) Fruity (eg, orange and pink) Nutty (eg, brown and yellow) Other (eg, purple)	—
Pack imagery	Flavor cued Flavor not cued	If flavor cued, classified into the following subtypes: Animal (eg, penguin and polar bear) Nature (eg, glacier and palm tree) Other (eg, switch button)	—
Others	Flavor cued Flavor not cued	If flavor cued, classified into the following subtypes: Cool or minty (eg, cuban coolness and cooling taste) Flavor (eg, max flavor and two tastes)	—
Number of flavor-cued elements	0 1 2 3 4 5	Sum the number of flavor-cued elements (responses)	—
Capsule imagery (stick)	Flavor cued Flavor not cued	If flavor cued, classified into the following subtypes: Cool or minty (eg, blue and green) Fruity (eg, orange and pink) Nutty (eg, brown and yellow) Other (eg, purple)	—
Flavoring method			
Inner wrapper	Flavored Not flavored	For each product, one team assessed the presence of flavoring using the orthonasal smelling method.	—
Cigarette wrapper	Flavored Not flavored	For each product, one team assessed the presence of flavoring using the orthonasal smelling method.	—
Tobacco leaves	Flavored Not flavored	For each product, one team assessed the presence of flavoring using the orthonasal smelling method.	—
Crushable capsule	Flavored Not flavored	For each product, one team assessed the presence of flavoring using the orthonasal smelling method.	—
Cellulose filter	Flavored Not flavored	For each product, one team assessed the presence of flavoring using the orthonasal smelling method.	—
Charcoal filter	Flavored Not flavored	For each product, one team assessed the presence of flavoring using the orthonasal smelling method.	—
Polylactic acid filter^f^	Flavored Not flavored	For each product, one team assessed the presence of flavoring using the orthonasal smelling method.	—
Hollow tube^f^	Flavored Not flavored	For each product, one team assessed the presence of flavoring using the orthonasal smelling method.	—
Others	Flavored Not flavored	For each product, one team assessed the presence of flavoring using the orthonasal smelling method.	—

a Only applicable for continuous data.

b Not available.

c All prices and currency values are reported in Korean won (KRW). The exchange rate as of November 2024 was approximately 1390 KRW to US $1.

d Not applicable for heated tobacco products.

e Flavor descriptors on tobacco packaging were coded based on prior tobacco packaging studies that typically distinguished menthol or mint, fruit or citrus, beverage, concept or functional, and other descriptors. Consistent with these studies, the further subdivision of flavor cue elements (eg, classifying blue and green as indicators of cool or minty) was informed by and closely aligned with prior studies.

f Only applicable for heated tobacco products.

For the stick-level flavor cue, we assessed the presence of capsule imagery on the stick surface. As illustrated in Figure S5 in Multimedia Appendix 1, capsule imagery was defined as a circular symbol resembling a crushable capsule printed on the stick. When present, it was classified using the same 4 color groups applied to pack color cues (ie, cool or minty, fruity, nutty, and others), based on the dominant color tone of the capsule symbol.

#### Sensory Measures

Sensory measures were collected to detect flavor additives and identify their location within the product. Orthonasal smelling [30], a validated method for detecting flavors through direct olfactory perception without inhalation [30,31], was used as a pragmatic screening method to detect the presence or absence of a perceptible nontobacco odor. This aligns with the definition of a “characterizing flavor” as a clearly noticeable smell or taste before or during consumption [44].

Using orthonasal smelling, each cigarette and HTP was assessed across 7 common components: inner wrapper, cigarette wrapper, tobacco leaves, crushable capsule, cellulose filter, charcoal filter, and others (eg, flavor card) [32]. For HTPs, 2 additional components (polylactic acid filter and hollow tube) were included [45]. Sticks were disassembled, and each component was evaluated separately. When the same odor was detectable in multiple components, the component with the strongest odor was recorded as the primary source. Because orthonasal smelling of tobacco products, even when unlit, may still involve minor sensory irritation or discomfort, all assessments were conducted in a ventilated room to minimize potential irritation and maintain olfactory sensitivity [46]. Coders also took breaks as needed to minimize olfactory fatigue and carryover effects before finalizing their judgments.

### Analysis

Descriptive analyses and data tabulation were conducted using Microsoft Excel. All characteristics were assessed according to tobacco product type (cigarettes vs HTPs). Categorical variables were presented as frequencies and percentages, while continuous variables were summarized using the minimum, median, mean, and maximum values. Continuous variables were additionally categorized, where applicable, to facilitate comparisons. Differences between cigarettes and HTPs were examined using chi-square tests in SAS (version 9.4; SAS Institute Inc), with a 2-sided significance level of *P*<.05. For variables with sparse expected cell counts (n<5), Fisher exact tests were used instead. For flavoring components, which allowed multiple responses, each component was treated as a binary variable (present vs absent). Proportional differences between cigarettes and HTPs were evaluated using separate chi-square tests.

### Ethical Considerations

This study did not involve human participants or animals. The data were collected from publicly available commercial tobacco products. Therefore, ethics approval was not required and was not sought.

## Results

### Sample General Characteristics

A total of 214 tobacco products were included in the final analytic sample, comprising 150 cigarettes and 64 HTPs. The descriptive characteristics of the collected products are provided in Table 2 and Table S3 in Multimedia Appendix 1. More than half of cigarettes (50%) and HTPs (51.6%) were manufactured by KT&G. The average tar and nicotine contents displayed on cigarette packs were 2.70 mg (range 0.1‐8.0 mg) and 0.24 mg (range: 0.01‐0.7 mg), respectively. The display of these contents is not mandatory for HTPs, and none of the HTP packs included them. All products complied with mandated health warnings.

**Table 2.** General characteristics of samples (N=214).

Variable	Cigarette (n=150), n (%)	Heated tobacco product (n=64), n (%)	*P* value^a^
Manufacturer			<.001
British American Tobacco (BAT)	21 (14)	10 (15.6)	
Japan Tobacco International (JTI)	26 (17.3)	0 (0)	
Philip Morris International Korea (PMIK)	28 (18.7)	21 (32.8)	
KT&G	75 (50)	33 (51.6)	
Price (KRW^b^)			<.001
<4500 KRW	19 (12.7)	0 (0)	
=4500 KRW	116 (77.3)	33 (51.6)	
>4500 KRW	15 (10)	31 (48.4)	
Number of sticks per pack			.70^c^
14	1 (0.7)	0 (0)	
20	149 (99.3)	64 (100)	
Tar content (mg)^d^			<.001
<2.7 mg	82 (54.7)	0 (0)	
≥2.7 mg	68 (45.3)	0 (0)	
N/A^e^	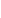 0 (0)	64 (100)	
Nicotine content (mg)^d^			<.001
<0.2 mg	79 (52.7)	0 (0)	
=0.2 mg	16 (10.7)	0 (0)	
>0.2 mg	55 (36.7)	0 (0)	
N/A^e^	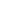 0 (0)	64 (100)	
Pack width (mm)			<.001
<60 mm	150 (100)	10 (15.6)	
≥60 mm	0 (0)	54 (84.4)	
Pack depth (mm)			<.001
<17 mm	58 (38.7)	63 (98.4)	
≥17 mm	92 (61.3)	1 (1.6)	
Pack height (mm)			<.001
<82 mm	0 (0)	55 (85.9)	
≥82 mm	150 (100)	9 (14.1)	
Pack shape			<.001
Rectangle	82 (54.7)	54 (84.4)	
Round-edged rectangle	45 (30)	3 (4.7)	
Half round-edged rectangle	23 (15.3)	7 (10.9)	
Stick length (mm)			<.001
<79 mm	150 (100)	55 (85.9)	
≥79 mm	0 (0)	9 (14.1)	
Health warning compliance			N/A^e^
Yes	150 (100)	64 (100)	
No	0 (0)	0 (0)	
Marketing text (excluding brand name)^f^			.03
Yes	103 (68.7)	34 (53.1)	
No	47 (31.3)	30 (46.9)	

a *P* values were obtained from chi-square tests comparing the distribution of characteristics between cigarettes and heated tobacco products.

b All prices and currency values are reported in Korean won (KRW). The exchange rate as of November 2024 was approximately 1390 KRW to US $1.

c The *P* value was calculated using the Fisher exact test.

d None of the heated tobacco product packs displayed nicotine or tar content.

e NA: not applicable.

f Marketing text includes “activated carbon filter,” “nano capsule technology,” and “smartcore induction system,” among others.

The main differences between cigarettes and HTPs were pack size (*P*<.001) and shape (*P*<.001) (Figure S6 in Multimedia Appendix 1). Cigarette sticks were longer, resulting in taller, vertically oriented rectangular packs with variation in depth. HTP packs were shorter, reflecting the shorter stick length, and had a consistent, landscape-oriented rectangular shape.

Although no distinct qualitative differences in promotional elements were observed between cigarettes and HTPs, a higher proportion of cigarette packs (68.7%) included marketing texts (*P*=.030) compared with HTPs (53.1%). Most of these statements highlighted hand and mouth odor reduction technology. For example, texts such as “activated carbon filter,” “nano capsule technology,” and “smartcore induction system” were frequently displayed, serving as marketing claims to emphasize the companies’ technological capabilities.

### Flavor Cue

The distribution of flavor cues on packaging is summarized in Table 3. Among the 5 components of pack-level flavor cues, pack color was the most common, appearing on 97 (64.7%) cigarettes and 61 (95.3%) HTPs (*P*<.001). Within pack color cues, cool or minty shades (blue, green, and sky blue) were most frequent, followed by fruity (red, pink, and orange), nutty (brown, dark brown, and yellow), and others (dark green and purple). The second most frequent cues varied by product type, but product names and capsule images were frequently used in both. Product names often implied coolness, such as “aqua,” “cool shot,” “frozen,” and “ice.” Capsule images, typically placed on the front of the pack, signaled flavor through colors associated with specific tastes. For example, blue tones for menthol or ice and red tones for cherry or berry. Pack imagery was less frequently used but featured cool- or minty-related images such as animals (eg, penguins) or natural scenes (eg, glaciers), reinforcing coolness, and other cues included promotional terms such as “double capsule” and “refreshing scent.” In addition, capsule imagery on the stick was observed in 71 (47.3%) cigarettes and 37 (57.8%) HTPs (*P*=.16). Among products with capsule imagery, cigarettes were predominantly cool or minty (59.2%), whereas HTPs were more evenly distributed across cool or minty (27%), fruity (29.7%), and other categories (27%).

**Table 3.** Flavor cues and flavoring methods according to tobacco product type.

Variable	Cigarettes (n=150), n (%)	Heated tobacco products (n=64), n (%)	*P* value
Flavor cues (pack)	101 (67.3)	61 (95.3)	<.001
Product name	61 (40.7)	44 (68.8)	<.001
Cool or minty	24 (39.3)	15 (34.1)	
Color	20 (32.8)	14 (31.8)	
Flavor	5 (8.2)	3 (6.8)	
Technology	6 (9.8)	7 (15.9)	
Other	6 (9.8)	5 (11.4)	
Pack color	97 (64.7)	61 (95.3)	<.001
Cool or minty	63 (64.9)	32 (52.5)	
Fruity	21 (21.6)	12 (19.7)	
Nutty	9 (9.3)	5 (8.2)	
Other	4 (4.1)	12 (19.7)	
Capsule imagery	70 (46.7)	39 (60.9)	.06
Cool or minty	41 (58.6)	18 (46.2)	
Fruity	20 (28.6)	14 (35.9)	
Nutty	4 (5.7)	1 (2.6)	
Other	5 (7.1)	6 (15.5)	
Pack imagery	22 (14.7)	9 (14.1)	.91
Animal	4 (18.2)	0 (0)	
Nature	16 (72.7)	6 (66.7)	
Other	2 (9.1)	3 (33.3)	
Other cues	21 (14)	13 (20.3)	.81
Cool or minty	11 (52.4)	9 (69.2)	
Flavor	10 (47.6)	4 (30.8)	
Number of flavor-cued elements^a^			<.001^b^
0	49 (32.7)	3 (4.7)	
1	11 (7.3)	3 (4.7)	
2	35 (23.3)	25 (39.1)	
3	33 (22)	22 (34.4)	
4	19 (12.7)	8 (12.5)	
5	3 (2)	3 (4.7)	
Capsule imagery (stick)	71 (47.3)	37 (57.8)	.16
Cool or minty	42 (59.2)	10 (27)	
Fruity	11 (15.5)	11 (29.7)	
Nutty	11 (15.5)	6 (16.2)	
Others	7 (9.8)	10 (27)	
Pack and stick flavor cue combination	150 (100)	64 (100)	<.001
Pack and stick cues	69 (46)	37 (57.8)	
Pack-only cues	32 (21.3)	24 (37.5)	
Stick-only cues	2 (1.3)	0 (0)	
Neither	47 (31.3)	3 (4.7)	
Flavored products^c^	89 (59.3)	54 (84.4)	<.001
Inner wrapper	16 (10.7)	8 (12.5)	.67
Cigarette wrapper	10 (6.7)	15 (23.4)	<.001
Tobacco leaves	18 (12)	29 (45.3)	<.001
Crushable capsule	73 (48.7)	37 (57.8)	.22
Cellulose filter	22 (14.7)	7 (10.9)	.47
Charcoal filter	1 (0.7)	0 (0)	≥.99
Polylactic acid filter^d^	—^e^	1 (1.6)	—
Hollow tube^d^	—	15 (23.4)	—
Others	11 (7.3)	2 (3.1)	.35
Number of flavoring methods^f^			<.001
0	61 (40.7)	10 (15.6)	
1	51 (34)	19 (29.7)	
2	19 (12.7)	17 (26.6)	
3	15 (10)	11 (17.2)	
4	4 (2.7)	7 (10.9)	
Products with crushable capsules^g^	73 (100)	37 (100)	<.001
Only a crushable capsule	40 (54.8)	17 (45.9)	
Crushable capsule + 1 method	16 (21.9)	13 (35.1)	
Crushable capsule + 2 methods	14 (19.2)	3 (8.2)	
Crushable capsule + 3 methods	3 (4.2)	4 (10.8)	
Flavoring methods used jointly with crushable capsules^h^			<.001
Inner wrapper	12 (36.4)	5 (25)	
Cigarette wrapper	3 (9.1)	3 (15)	
Tobacco leaves	13 (39.4)	14 (70)	
Cellulose filter	17 (51.5)	4 (20)	
Charcoal filter	0 (0)	0 (0)	
Polylactic acid filter	—	1 (5)	
Hollow tube	—	3 (15)	
Other	8 (24.2)	1 (5)	

a Total count of all flavor cues identified on the pack.

b *P* values were calculated using the Fisher exact test where applicable.

c Percentages may sum to more than 100% because categories are not mutually exclusive; a single product may contain flavor additives in multiple components.

d Applicable only to heated tobacco products.

e Not applicable.

f Total count of components with detectable flavor additives among products.

g Percentages are calculated among products with crushable capsules.

h Total count of components with detectable flavor additives among products containing a crushable capsule combined with at least 1 additional flavoring method.

**Figure 2.**
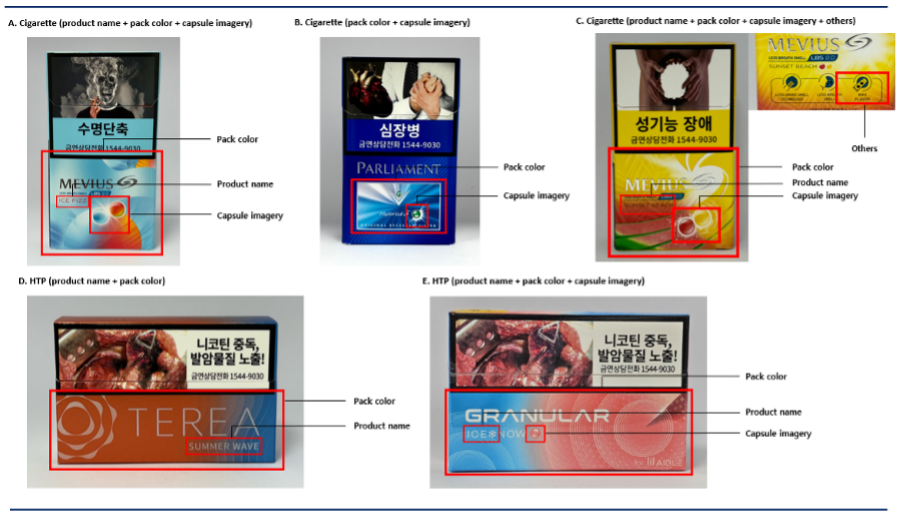
Common combinations of flavor cues in cigarettes and heated tobacco products.

Figure 2 presents examples of flavor cue combinations by product. Overall, 101 (67.3%) cigarettes and 61 (95.3%) HTPs included at least 1 flavor cue (*P*<.001). For cigarettes, color and capsule images were commonly used together, with the most frequent combinations being “product name + color + capsule image” (11.3%; Figure 2A) and “color + capsule image” (10.7%; Figure 2B). All HTPs with flavor cues included pack color as one of the cue elements, often reinforced by additional cues such as product name and capsule imagery (Figures 2D and 2E).

### Flavoring Method

Of the 214 products analyzed, 143 (66.8%) contained at least 1 flavoring component (Table 3). Flavoring was identified in 89 (59.3%) cigarettes and 54 (84.4%) HTPs (*P*<.001). Crushable capsules were the most common flavoring method in both cigarettes (48.7%) and HTPs (57.8%) (*P*=.22). In cigarettes, flavor additives were also found in cellulose filters (14.7%) and tobacco leaves (12%). In contrast, HTPs used a wider range of flavoring methods, including tobacco leaves (45.3%), cigarette wrappers (23.4%), and hollow tubes (23.4%).

Although up to 9 flavoring components (7 for cigarettes) were possible, both cigarettes and HTPs showed a maximum of 4 components per product. Overall, 25.4% of cigarettes and 54.7% of HTPs used 2 or more flavoring methods. Among products with crushable capsules, 54.8% of cigarettes and 45.9% of HTPs used capsules as the sole flavoring method. In both product types, up to 3 additional components were combined with capsules. Among capsule-containing products that used at least 1 additional flavoring component, the most common paired components were cellulose filters (51.5%) and tobacco leaves (39.4%) in cigarettes. For HTPs, tobacco leaves (70%) and inner wrappers (25%) were the most frequently paired. The distribution of paired components differed significantly between cigarettes and HTPs (*P*<.001), with a higher proportion of HTPs containing flavoring in tobacco leaves (70%) compared with cigarettes (39.4%).

**Figure 3.**
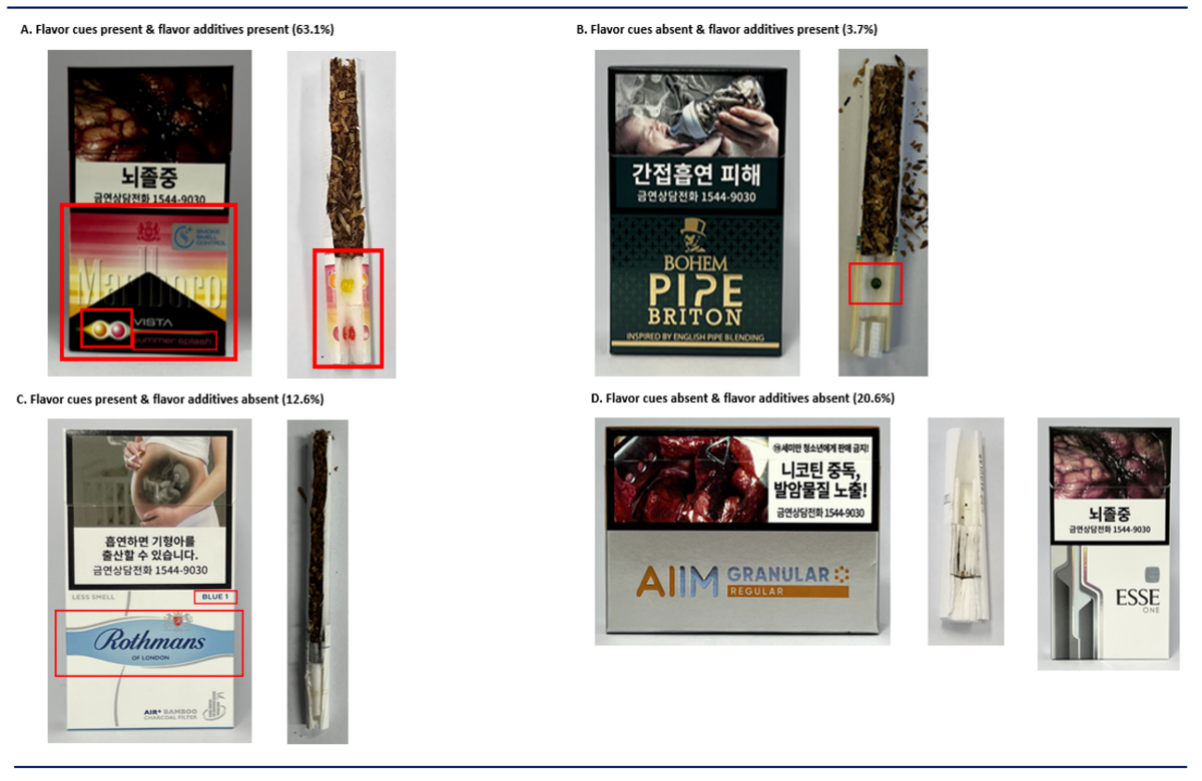
Comparison of flavor cues and actual flavoring in tobacco products.

Across all products analyzed, 63.1% (54% of cigarettes and 84.4% of HTPs) both had flavor cues on the pack and contained flavor additives (Figure 3A), while 3.7% (5.3% of cigarettes and 0% of HTPs) contained flavor additives without any packaging cues (Figure 3B). Conversely, 12.6% (13.3% of cigarettes and 10.9% of HTPs) displayed cues but lacked detectable flavor additives (Figure 3C), and 20.6% (27.3% of cigarettes and 4.7% of HTPs) showed neither cues nor additives (Figure 3D).

## Discussion

### Principal Findings and Implications

We conducted systematic surveillance of cigarettes and HTPs sold in Korea, integrating pack- and stick-level coding of flavor cues with component-based assessment of flavor additives. Our analysis showed that pack sizes and shapes were not standardized and varied across both product types. Pack color was the most common flavor cue, followed by capsule imagery and product names, often used in combination. Most products used 2-3 cues simultaneously, reinforcing flavor signals. Flavor cues and additives were more prevalent among HTPs than cigarettes. Although capsules were the dominant flavoring method, additives were also incorporated into other components.

The flavor cues identified align with previous tobacco pack surveillance studies documenting widespread use of packaging elements to signal flavor. However, regulatory environments shape how these cues manifest. In markets without explicit restrictions, such as the Philippines, 58.5% of tobacco products contained flavor cues through direct descriptors beneath the product name [11]. In contrast, Korea’s Article 9‐3 of the NHPA prohibits explicit flavor descriptors such as “menthol” or “cherry,” including text or imagery, on tobacco packaging [27]. Nevertheless, flavoring continued to be implied through indirect marketing strategies, using terms such as “Ice” or “Tropical” and capsule imagery on both packs and sticks. Consequently, the prevalence of flavor cues among the products collected in Korea (75.7%) was far higher than the average among countries without flavor bans (22.7%) [12]. These findings suggest that regulatory approaches limited to explicit flavor indicators may be insufficient to eliminate flavor-related marketing.

A key contribution of this study is the combined assessment of flavor cues across both packs and sticks, alongside component-based screening of flavoring additives. Over 60% of products combined flavor cues with detectable flavor additives, but cues were also present in products without detectable flavor additives. Pack-level cues were often reinforced at the point of use, as 47% of cigarettes and 58% of HTPs featured capsule imagery on the stick. This pattern highlights that flavor-related appeal can be generated independently or synergistically through perceptual mechanisms (pack and stick design) [1,47] and material product features (additives) [48]. Linking consumer-facing signals with product-level evidence, therefore, provides a more comprehensive characterization of flavored tobacco products.

These findings have practical implications for enforcing flavor ban policies. Comprehensive flavor bans alone may be insufficient, as they leave room for circumvention. Even without flavor additives, packaging elements that imply flavor can shape perceptions of taste, quality, and harm [41] and may even modulate craving-related neural responses, reinforcing product appeal [49]. Moreover, in jurisdictions where flavor bans have been implemented without plain packaging, the industry has introduced pack- and stick-level design features that insinuate flavor characteristics despite the absence of explicit additives [50]. Flavor bans and plain packaging covering both packs and sticks should therefore be implemented in tandem. However, enforcing flavor bans can be technically complex due to ambiguity in defining “characterizing flavor” [51] and limited standardized chemical testing capacity [52]. In this context, plain packaging may serve as a more immediately implementable strategy while regulatory systems strengthen enforcement capacity for flavor bans.

Several areas for future research follow from our findings. The high prevalence of flavoring additives among sampled products is broadly consistent with reported sales patterns of flavored tobacco products in Korea [26], highlighting a discrepancy between market patterns and the relatively lower self-reported use of flavored tobacco noted in Korea [53]. Only 29.8% of Korean smokers reported using flavored tobacco products (excluding capsule products), whereas only 26.7% reported that their usual brand contained a flavor capsule [26], which is lower than both the market share of flavored products and the prevalence of additives detected in our assessment. Our findings show that while crushable capsules were common, flavor additives were also frequently incorporated into less visible components such as tobacco leaves, wrappers, and internal filters. Because flavoring in these components is less noticeable and not widely recognized as a marker of flavored tobacco, such products may go unrecognized by consumers as flavored. Future research could examine whether this gap reflects underrecognition of these less salient forms of flavor delivery, or other factors such as higher consumption intensity among flavored-product users [54]. Additionally, the high proportion of discontinued products identified from our sampling procedures suggests rapid market turnover. Continued surveillance research will therefore be important to track evolving product design and flavoring strategies.

Given that evidence on HTP flavoring remains limited globally [55], our findings provide early empirical evidence in this area. Compared with cigarettes, HTPs use more pervasive cues and diverse flavoring methods, often using multiple flavoring methods across various components rather than being limited to capsules alone. As HTP markets continue to expand [56], surveillance frameworks developed for cigarettes, including pack- and stick-level assessment and evaluation of flavor additives, should be extended to HTPs. Our methods and findings offer a preliminary framework that may be applied in other settings to systematically assess the characteristics of HTPs.

Another global implication of our study is that the patterns documented in Korea may extend beyond the domestic market. KT&G, a major Korean tobacco manufacturer and global exporter [57-60], has reported increasing exports of flavored products and capsules [59], with packaging designs in some export markets that are identical or highly similar to those sold in Korea [60,61]. Surveillance of products sold in Korea may therefore provide an early signal of evolving industry tactics that could emerge in other jurisdictions.

### Limitations

Our study has several limitations. First, products were purchased in one metropolitan area and primarily from major convenience store chains, so availability may differ in other regions or retail channels (eg, supermarkets and duty-free outlets). Second, some identified products could not be collected due to discontinuation or low demand, which may have introduced selection bias and affected estimates of flavor cues and additives. For example, niche or experimental flavored variants are more likely to be dropped from retail shelves, potentially leading to an underestimation of the diversity or prevalence of flavor-related features. Third, coding was conducted through team-based consensus rather than independent double coding, which may have introduced bias and precluded calculation of intercoder reliability. Fourth, our sensory screening relied on a pragmatic, nonblinded orthonasal assessment conducted by trained research staff rather than professional sensory analysts. Subtle additives may have been missed, and component attribution should be interpreted as approximate due to potential odor diffusion. Future work should incorporate blinded sensory testing conducted by expert sensory panelists and chemical analyses to improve the detection and localization of flavoring additives.

### Conclusions

To our knowledge, this study is among the first to simultaneously examine flavor cues on cigarette and HTP packaging and sticks, alongside the presence of flavor additives. By integrating pack- and stick-level assessments, we provide evidence on how flavor cues and additives work together to enhance product appeal. Our findings show that flavoring in tobacco products extends beyond visible pack design or crushable capsules to multiple structural components, highlighting the need for regulations that address both perceptual mechanisms and sources of flavoring. Incorporating stick-level monitoring and HTPs into existing surveillance systems will be crucial for identifying concealed flavoring cues and methods. These efforts can strengthen the implementation of World Health Organization (WHO) FCTC Articles 9 and 10 (regulation and disclosure of tobacco product contents and emissions) and Article 11 (packaging and labeling).

## Supplementary material

10.2196/87537 Multimedia Appendix 1 : Additional tables and figures.

## Abbreviations

FCTC: Framework Convention on Tobacco Control
HTP: heated tobacco product
NHPA: National Health Promotion Act
TPackSS: Tobacco Pack Surveillance System
WHO: World Health Organization
