# Supplementary material for: Systematic Assessment of Flavor Cues and Additives in Cigarettes and Heated Tobacco Products in Korea: Cross-Sectional Surveillance Study
Source: JMIR Public Health Surveill. 2026 May 25;12:e87537. doi: 10.2196/87537 (PMC13200799; doi:10.2196/87537)

**Table S1. Cigarette Product Sample List**

**Table S2. Heated Tobacco Product Sample List**

**Table S3. General Characteristics of Samples (N=214)**

**Figure S1. Tobacco Product Photography Guideline (Before Opening Tobacco Product Pack)**

**Figure S2. Tobacco Product Photography Guideline (After Opening Tobacco Product Pack)**

**Figure S3. Components of Flavor Cues**

**Figure S4. Components of Tobacco Products**

**Figure S5. Example of stick-level capsule imagery and color-group classification**

**Figure S6. Comparison of Tobacco Product Pack by Dimension**

**Table S1. Cigarette Product Sample List**

| **No.** | **Brand** | **ITC KRA3**  **(2023)** | **Ministry of Health and Welfare (2023)** | **Obtained /**  **Reason not obtained** |
| --- | --- | --- | --- | --- |
| 1 | ESSE CHANGE 1mg | O | O | Obtained |
| 2 | ESSE CHANGE 4mg | O | O | Obtained |
| 3 | ESSE CHANGE Frozen 1mg | O | O | Obtained |
| 4 | ESSE CHANGE Shooting RED | X | X | Obtained |
| 5 | ESSE CHANGE Himalaya | O | O | Obtained |
| 6 | ESSE CHANGE Himalaya Winter | X | X | Obtained |
| 7 | ESSE CHANGE W 1mg | O | O | Obtained |
| 8 | ESSE CHANGE Up 1 mg | O | O | Obtained |
| 9 | ESSE CHANGE LiNN | O | O | Obtained |
| 10 | ESSE CHANGE Bing | X | O | Obtained |
| 11 | ESSE CHANGE Gram | O | O | Obtained |
| 12 | ESSE CHANGE Coolips | X | O | Obtained |
| 13 | ESSE CHANGE Double | X | O | Discontinued |
| 14 | ESSE CHANGE Secret | X | O | Discontinued |
| 15 | ESSE CHANGE Icefall | X | O | Discontinued |
| 16 | ESSE PRIME | O | O | Obtained |
| 17 | ESSE GOLDEN LEAF | O | X | Obtained |
| 18 | ESSE GOLDEN LEAF 0.5mg | O | X | Obtained |
| 19 | ESSE GOLDEN LEAF 1mg | O | O | Obtained |
| 20 | ESSE GOLDEN LEAF 3mg | X | O | Low-demand |
| 21 | ESSE ROYAL PALACE | X | O | Low-demand |
| 22 | ESSE ICE 1mg | O | O | Obtained |
| 23 | ESSE ICE (MENTHOL) | O | O | Obtained |
| 24 | ESSE ONE | O | O | Obtained |
| 25 | ESSE SPECIAL GOLD 3.5mg | O | O | Obtained |
| 26 | ESSE SPECIAL GOLD 0.5mg | O | O | Obtained |
| 27 | ESSE SPECIAL GOLD 1mg | O | O | Obtained |
| 28 | ESSE SPECIAL GOLD OCEAN | X | O | Low-demand |
| 29 | ESSE SOO | O | O | Obtained |
| 30 | ESSE SOO 0.5mg | O | O | Obtained |
| 31 | ESSE SOO 0.1mg | O | O | Obtained |
| 32 | ESSE SOO MASTERPIECE | X | O | Discontinued |
| 33 | ESSE PRESSO 1mg | O | O | Obtained |
| 34 | ESSE it's Deep Brown | O | O | Obtained |
| 35 | ESSE it's Bubble Purple | X | O | Discontinued |
| 36 | ESSE Sense 1mg | O | O | Obtained |
| 37 | ESSE Classic | O | O | Low-demand |
| 38 | ESSE EDGE | O | O | Obtained |
| 39 | ESSE EDGE 5mg | O | X | Discontinued |
| 40 | ESSE EDGE ICEPOINT 1mg | O | X | Discontinued |
| 41 | BOHEM PIPE BRITON | X | X | Obtained |
| 42 | BOHEM CIGAR NO.1 | O | O | Obtained |
| 43 | BOHEM CIGAR NO.3 | O | O | Obtained |
| 44 | BOHEM CIGAR NO.6 | O | O | Obtained |
| 45 | BOHEM CIGAR MASTER | O | O | Obtained |
| 46 | BOHEM CIGAR MINI 1mg | O | O | Obtained |
| 47 | BOHEM CIGAR MINI 5mg | O | O | Obtained |
| 48 | BOHEM CIGAR SLIM FIT BROWN 1mg | O | O | Obtained |
| 49 | BOHEM CIGAR SLIM FIT WHITE 1mg | O | O | Obtained |
| 50 | BOHEM CIGAR SLIM SKINNY | X | O | Discontinued |
| 51 | BOHEM CIGAR LIBRE | O | O | Obtained |
| 52 | BOHEM CIGAR LIBRE X1 | X | O | Discontinued |
| 53 | BOHEM CIGAR PIPE SCOTTIE | O | O | Obtained |
| 54 | BOHEM CIGAR CARIBE | O | O | Obtained |
| 55 | BOHEM CIGAR ICE FIT | O | O | Obtained |
| 56 | BOHEM CIGAR CUBANA DOUBLE | O | O | Obtained |
| 57 | BOHEM CIGAR CUBANA 1mg | O | O | Obtained |
| 58 | BOHEM CIGAR CUBANA 17+ DOUBLE | O | O | Discontinued |
| 59 | BOHEM CIGAR CUBANA 17+ NO.1 | O | O | Discontinued |
| 60 | BOHEM CIGAR CUBANA 17+ NO.6 | O | O | Discontinued |
| 61 | BOHEM CIGAR MINI Roast | X | O | Discontinued |
| 62 | Raison Hyvaa Ice Tundra | X | X | Obtained |
| 63 | Raison Black | O | O | Obtained |
| 64 | Raison Blue | O | O | Obtained |
| 65 | Raison aero 1mg | O | O | Discontinued |
| 66 | Raison Ice Presso | O | O | Obtained |
| 67 | Raison Sun Presso | O | O | Obtained |
| 68 | Raison French Black | O | O | Obtained |
| 69 | Raison French Yogurt | O | O | Obtained |
| 70 | Raison French Hyvaa | O | O | Obtained |
| 71 | Raison French Polar | X | O | Discontinued |
| 72 | Raison French Ssom | O | O | Obtained |
| 73 | Raison French Cleo | O | O | Obtained |
| 74 | Raison French Burn | X | O | Discontinued |
| 75 | Raison French Line | X | O | Discontinued |
| 76 | Raison French Ice Blan | X | O | Obtained |
| 77 | Raison green | O | X | Discontinued |
| 78 | Raison presso 1mg | O | O | Discontinued |
| 79 | Raison presso 6mg (café) | O | X | Discontinued |
| 80 | Raison iONIA ISLAND PINK | X | X | Obtained |
| 81 | Raison iONIA AQUA GREEN | X | X | Obtained |
| 82 | Raison RESERVE | X | O | Low-demand |
| 83 | THIS ORIGINAL | O | O | Obtained |
| 84 | THIS PLUS | O | O | Obtained |
| 85 | THIS AFRICA GOLA | X | O | Discontinued |
| 86 | THIS AFRICA LULA | O | O | Obtained |
| 87 | THIS AFRICA MOLA | O | O | Obtained |
| 88 | THIS AFRICA SUNDAY | X | O | Discontinued |
| 89 | THIS AFRICA ICE JACK | O | O | Obtained |
| 90 | THIS AFRICA ICE KULA | X | O | Discontinued |
| 91 | THIS AFRICA Hai hai | X | O | Discontinued |
| 92 | THE ONE BLUE 1.0mg | O | O | Obtained |
| 93 | THE ONE SKY | X | O | Discontinued |
| 94 | THE ONE ORANGE 0.5mg | O | O | Obtained |
| 95 | THE ONE WHITE 0.1mg | O | O | Obtained |
| 96 | THE ONE CHANGE 1.0mg | O | O | Obtained |
| 97 | THE ONE CHANGE LipToc 1.0mg | X | O | Discontinued |
| 98 | THE ONE IMPACT 1.0mg | O | O | Obtained |
| 99 | CLOUD NINE 1mg | O | O | Obtained |
| 100 | CLOUD NINE 5mg | O | O | Obtained |
| 101 | SIMPLE ACE 1mg | O | O | Obtained |
| 102 | SIMPLE ACE 5mg | O | O | Discontinued |
| 103 | SIMPLE CLASSIC | O | O | Obtained |
| 104 | SIMPLE VISION | X | O | Discontinued |
| 105 | HALLASAN | O | O | Obtained |
| 106 | SEASONS | O | O | Obtained |
| 107 | TONINO LAMBORGHINI ICEVOLT GT | O | O | Obtained |
| 108 | TONINO LAMBORGHINI ICEVOLT | X | O | Discontinued |
| 109 | TONINO LAMBORGHINI ICE TORDADO | O | X | Discontinued |
| 110 | TIMELESS TIME MID | O | O | Obtained |
| 111 | TIMELESS TIME CLASSIC | X | O | Discontinued |
| 112 | TIMELESS TIME HUMMING | X | O | Discontinued |
| 113 | 88 ReturnS | X | O | Discontinued |
| 114 | DAVIDOFF CLASSIC | X | O | Discontinued |
| 115 | DAVIDOFF BLUE | X | O | Discontinued |
| 116 | ENTZ 3.5mg | O | O | Discontinued |
| 117 | LILAC | O | O | Low-demand |
| 118 | ROSE | O | X | Discontinued |
| 119 | MARLBORO SILVER | O | X | Obtained |
| 120 | MARLBORO GOLD | O | O | Obtained |
| 121 | MARLBORO GOLD TOUCH | O | O | Discontinued |
| 122 | MARLBORO RED | O | O | Obtained |
| 123 | MARLBORO MEDIUM | O | O | Obtained |
| 124 | MARLBORO BLACK FRESH | O | O | Obtained |
| 125 | MARLBORO WHITE FRESH | O | O | Obtained |
| 126 | MARLBORO ICE BLAST | O | O | Obtained |
| 127 | MARLBORO ICE BLAST 1mg | O | O | Obtained |
| 128 | MARLBORO FLAVOR PLUS | X | O | Discontinued |
| 129 | MARLBORO HYBRID 1mg | O | O | Obtained |
| 130 | MARLBORO HYBRID 5mg | O | O | Obtained |
| 131 | MARLBORO ZING FUSION | O | X | Discontinued |
| 132 | MARLBORO ZING FUSION DOUBLE | X | O | Discontinued |
| 133 | MARLBORO ZERO ADDITIVES | X | O | Discontinued |
| 134 | MARLBORO SHUFFLE | X | O | Discontinued |
| 135 | MARLBORO VISTA GARDEN SPLASH | X | X | Obtained |
| 136 | MARLBORO VISTA SUMMER SPLASH | X | O | Obtained |
| 137 | MARLBORO VISTA BLOSSOM MIST | X | X | Obtained |
| 138 | MARLBORO VISTA TROPICAL SPLASH | O | O | Obtained |
| 139 | MARLBORO VISTA TROPICAL BREEZE | O | O | Obtained |
| 140 | MARLBORO VISTA FOREST MIST | O | O | Obtained |
| 141 | PARLIAMENT ONE | O | O | Obtained |
| 142 | PARLIAMENT SUPER SLIM ONE | O | O | Obtained |
| 143 | PARLIAMENT SUPER SLIM BLUE | O | O | Discontinued |
| 144 | PARLIAMENT SUPER SLIM RED | O | O | Obtained |
| 145 | PARLIAMENT AQUA 3 | O | O | Obtained |
| 146 | PARLIAMENT AQUA 5 | O | O | Obtained |
| 147 | PARLIAMENT HYBRID | O | O | Obtained |
| 148 | PARLIAMENT HYBRID ONE | X | O | Discontinued |
| 149 | PARLIAMENT HYBRID 1mg SUPERSLIM | O | O | Low-demand |
| 150 | PARLIAMENT HYBRID 5mg | O | O | Obtained |
| 151 | PARLIAMENT CLEAR FINISH | O | O | Obtained |
| 152 | PARLIAMENT TWIST | X | O | Discontinued |
| 153 | PARLIAMENT HYBRID TROPIC 5mg | O | X | Discontinued |
| 154 | PARLIAMENT DUAL SENSATION | X | O | Discontinued |
| 155 | PARLIAMENT DOUBLE WAVE | X | O | Discontinued |
| 156 | VIRGINIA SLIMS ONE | O | O | Obtained |
| 157 | VIRGINIA SLIMS BLUE | O | O | Obtained |
| 158 | VIRGINIA SLIMS GOLD | O | O | Obtained |
| 159 | LARK PREMIUM ONE (HARMONY) | O | O | Obtained |
| 160 | DUNHILL 1mg | O | O | Obtained |
| 161 | DUNHILL 1mg (14 cig) | O | O | Obtained |
| 162 | DUNHILL 3mg | O | O | Obtained |
| 163 | DUNHILL 6mg | O | O | Obtained |
| 164 | DUNHILL 6mg (14 cig) | X | O | Discontinued |
| 165 | DUNHILL SWITTCH PLUS ONE | O | O | Obtained |
| 166 | DUNHILL SWITCH PLUS 6mg | O | O | Obtained |
| 167 | DUNHILL FROST | O | O | Obtained |
| 168 | DUNHILL FINE CUT 1mg | O | O | Obtained |
| 169 | DUNHILL FINE CUT 0.1mg | O | O | Obtained |
| 170 | DUNHILL FINE CUT SUPREME 4mg | O | O | Obtained |
| 171 | DUNHILL FINE CUT FROST 1mg | O | O | Obtained |
| 172 | DUNHILL FINE CUT SWITCH | O | O | Obtained |
| 173 | DNUHILL FINE CUT MASTER 1mg | O | O | Discontinued |
| 174 | DNUHILL FINE CUT MASTER 3mg | O | X | Discontinued |
| 175 | DUNHILL FINE CUT TROPICAL CRUSH | O | O | Obtained |
| 176 | DUNHILL FINE CUT SMOOTH CRUSH INTRO | X | O | Discontinued |
| 177 | DUNHILL FINE CUT SMOOTH CRUSH | X | O | Discontinued |
| 178 | DUNHILL FINE CUT SMOOTH CRUSH NEW | O | O | Obtained |
| 179 | DUNHILL FINE CUT MELLOW CRUSH | X | O | Discontinued |
| 180 | DUNHILL FINE CUT MELLOW CRUSH NEW | O | O | Obtained |
| 181 | DUNHILL FINE CUT ELECTRIC CRUSH | X | O | Discontinued |
| 182 | DUNHILL FINE CUT ELECTRIC CRUSH NEW | X | O | Discontinued |
| 183 | DUNHILL RUBY BOOST | X | O | Discontinued |
| 184 | DUNHILL REMIX | X | O | Discontinued |
| 185 | DUNHILL DOLCE | O | O | Discontinued |
| 186 | DUNHILL ICE CUBE | O | O | Discontinued |
| 187 | DUNHILL CRISP | O | X | Discontinued |
| 188 | DUNHILL ORIENT | O | X | Discontinued |
| 189 | DUNHILL TOP LEAF | O | X | Discontinued |
| 190 | DUNHILL SUMMER CRUSH | X | O | Discontinued |
| 191 | DUNHILL EXOTIC CRUSH | X | O | Discontinued |
| 192 | DUNHILL EXOTIC CRUSH NEW | O | O | Discontinued |
| 193 | DUNHILL ALPS BOOST | O | O | Discontinued |
| 194 | DUNHILL RAINBOW BOOST | X | O | Duty-free only |
| 195 | DUNHILL COOL ONE | O | X | Discontinued |
| 196 | DUNHILL COOL 6mg | O | X | Discontinued |
| 197 | KENT PURPLE | O | O | Obtained |
| 198 | KENT SUPER SLIM 0.5mg | X | O | Obtained |
| 199 | KENT SUPER SLIM 1mg | X | O | Discontinued |
| 200 | KENT SUPER SLIM WHITE | X | O | Obtained |
| 201 | KENT BLUE | O | O | Obtained |
| 202 | KENT SILVER | O | X | Discontinued |
| 203 | KENT SWITCH | O | O | Obtained |
| 204 | KENT CLICK | O | O | Obtained |
| 205 | KENT CONVERTIBLES | O | X | Discontinued |
| 206 | KENT DOUBLE FRESH INTRO | X | O | Discontinued |
| 207 | KENT DOUBLE FRESH | O | O | Discontinued |
| 208 | MEVIUS ORIGINAL | O | O | Obtained |
| 209 | MEVIUS ONE | X | O | Discontinued |
| 210 | MEVIUS SKY BLUE | O | O | Obtained |
| 211 | MEVIUS SKY BLUE SUPER SLIM ONE | X | O | Discontinued |
| 212 | MEVIUS SKY BLUE SOFT | X | O | Obtained |
| 213 | MEVIUS SKY BLUE LONGS | X | O | Obtained |
| 214 | MEVIUS WIND BLUE | O | O | Obtained |
| 215 | MEVIUS E-STYLE 3mg | O | O | Obtained |
| 216 | MEVIUS E-STYLE 6mg | O | O | Obtained |
| 217 | MEVIUS LSS V5 | X | O | Discontinued |
| 218 | MEVIUS LSS ONE | O | O | Obtained |
| 219 | MEVIUS LSS WIND BLUE | O | O | Obtained |
| 220 | MEVIUS LSS SELECT | X | O | Discontinued |
| 221 | MEVIUS LBS PURPLE (OPTION2) | O | O | Obtained |
| 222 | MEVIUS LBS YELLOW | X | O | Obtained |
| 223 | MEVIUS LBS YELLOW 3mg | O | O | Obtained |
| 224 | MEVIUS LBS YELLOW SUPER SLIM | O | O | Obtained |
| 225 | MEVIUS LBS YELLOW MAX | O | O | Obtained |
| 226 | MEVIUS LBS YELLOW MAX SUPER SLIM | X | O | Discontinued |
| 227 | MEVIUS LBS BLUE | X | O | Discontinued |
| 228 | MEVIUS LBS BLUE SUPER SLIM | X | O | Discontinued |
| 229 | MEVIUS LBS MIX GREEN | X | O | Discontinued |
| 230 | MEVIUS LBS MIX GREEN SUPER SLIM | O | O | Obtained |
| 231 | MEVIUS LBS TROPICAL MIX 3mg | O | O | Obtained |
| 232 | MEVIUS LBS TROPICAL MIX 5mg | X | O | Discontinued |
| 233 | MEVIUS LBS SUNSET BEACH | X | O | Obtained |
| 234 | MEVIUS LBS SPARKLING MAX | X | O | Discontinued |
| 235 | MEVIUS LBS SPARKLING DEW | X | O | Obtained |
| 236 | MEVIUS LBS CITRO WAVE | X | X | Obtained |
| 237 | MEVIUS LBS ICE STORM | X | O | Obtained |
| 238 | MEVIUS LBS BANA SUPER SLIM | X | O | Discontinued |
| 239 | MEVIUS LBS ICE BANA | X | O | Obtained |
| 240 | MEVIUS LBS ICE FIZZ | X | O | Obtained |
| 241 | MEVIUS LBS LONG ISLAND | X | O | Obtained |
| 242 | CAMEL FILTERS | O | O | Obtained |
| 243 | CAMEL BLUE | O | O | Obtained |
| 244 | CAMEL SUPER SLIM 1mg | X | O | Obtained |
| 245 | CAMEL SUPER SLIM 3mg | X | O | Discontinued |
| 246 | CAMEL SILVER | O | X | Discontinued |
| 247 | LD MELODY SUPER SLIM | X | O | Low-demand |
| 248 | LD MELODY 1mg | X | O | Low-demand |
| 249 | LD XYLO 1mg | X | O | Low-demand |
| 250 | LD WAIKIKI DUO 1mg | X | O | Low-demand |
| 251 | LD BLUE LONGS 1mg | X | O | Low-demand |
| 252 | LD FRESH SUPER SLIM | X | O | Low-demand |
| 253 | NATURAL AMERICAN SPIRIT 3mg | X | O | Discontinued |
| 254 | NATURAL AMERICAN SPIRIT 6mg | X | O | Discontinued |

**Table S2. Heated Tobacco Product (HTP) Sample List**

| **No.** | **Brand** | **ITC KRA3 (2023)** | **Ministry of Health and Welfare (2023)** | **Obtained /**  **Reason not obtained** |
| --- | --- | --- | --- | --- |
| 1 | FIIT CHANGE | O | O | Obtained |
| 2 | FIIT CHANGE UP | O | O | Obtained |
| 3 | FIIT CHANGE Q | O | O | Obtained |
| 4 | FIIT CHANGE W | X | O | Discontinued |
| 5 | FIIT CHANGE UNIQ | X | X | Obtained |
| 6 | FIIT CHANGE TOK | O | O | Discontinued |
| 7 | FIIT MATCH | O | X | Discontinued |
| 8 | FIIT GOLDEN PIPE | O | O | Discontinued |
| 9 | FIIT SPARKY | O | O | Obtained |
| 10 | FIIT COOL SHOT | O | O | Obtained |
| 11 | FIIT ICE+ING | O | O | Obtained |
| 12 | FIIT ICE+EST | O | O | Obtained |
| 13 | MIIX MIX | O | O | Obtained |
| 14 | MIIX SHINE | O | O | Discontinued |
| 15 | MIIX COMBO | O | O | Obtained |
| 16 | MIIX UPTOO | X | X | Obtained |
| 17 | MIIX BLUSOME | X | X | Obtained |
| 18 | MIIX ORASOME | X | X | Obtained |
| 19 | MIIX ICE | O | O | Obtained |
| 20 | MIIX ICEAN | X | O | Obtained |
| 21 | MIIX ICE DOUBLE | O | O | Obtained |
| 22 | MIIX ICE ALOHA | O | O | Discontinued |
| 23 | MIIX ICE FRENCH | O | O | Obtained |
| 24 | MIIX ICE BANG | O | O | Obtained |
| 25 | MIIX CLASSY | O | O | Obtained |
| 26 | MIIX PRESSO | O | O | Discontinued |
| 27 | AIIM GRANULAR REGULAR | O | O | Obtained |
| 28 | AIIM GRANULAR BLUMING | X | X | Obtained |
| 29 | AIIM GRANULAR CUPPLE | X | X | Obtained |
| 30 | AIIM GRANULAR TWICE | O | O | Obtained |
| 31 | AIIM GRANULAR ICE | O | O | Obtained |
| 32 | AIIM GRANULAR ICE SNOW | X | X | Obtained |
| 33 | AIIM GRANULAR ICE RUSH | X | O | Obtained |
| 34 | AIIM REAL REGULAR | X | O | Obtained |
| 35 | AIIM REAL CIGARISH | X | X | Obtained |
| 36 | AIIM REAL CAMEO | X | O | Obtained |
| 37 | AIIM REAL SUNEST | X | X | Obtained |
| 38 | AIIM REAL ICE | O | O | Obtained |
| 39 | AIIM REAL ICE PEAK | X | X | Obtained |
| 40 | AIIM VAPOR STICK ICE DOUBLE | O | O | Obtained |
| 41 | AIIM VAPOR STICK CRASH | X | O | Discontinued |
| 42 | TEREA BLACK RUBY | X | X | Obtained |
| 43 | TEREA BLACK PURPLE | O | O | Obtained |
| 44 | TEREA BLACK GREEN | O | O | Obtained |
| 45 | TEREA BLACK YELLOW | X | O | Obtained |
| 46 | TEREA BLUE | O | O | Obtained |
| 47 | TEREA SILVER | O | O | Obtained |
| 48 | TEREA GREEN | O | O | Obtained |
| 49 | TEREA GREEN ZING | X | O | Obtained |
| 50 | TEREA AMBER | O | O | Obtained |
| 51 | TEREA YUGEN | O | O | Obtained |
| 52 | TEREA RUSSET | X | X | Obtained |
| 53 | TEREA TEAK | X | X | Obtained |
| 54 | TEREA SUMMER WAVE | O | O | Obtained |
| 55 | TEREA PURPLE WAVE | O | O | Obtained |
| 56 | TEREA ARBOR PEARL | X | X | Obtained |
| 57 | TEREA STARLING PEARL | X | X | Obtained |
| 58 | TEREA OASIS PEARL | X | X | Obtained |
| 59 | TEREA SUN PEARL | X | X | Obtained |
| 60 | HEETS AMBER LABEL | O | O | Discontinued |
| 61 | HEETS PURPLE LABEL | O | O | Discontinued |
| 62 | HEETS YELLOW LABEL | O | X | Discontinued |
| 63 | HEETS BLUE LABEL | O | O | Discontinued |
| 64 | HEETS GREEN LABEL | X | O | Obtained |
| 65 | HEETS GREEN ZING | O | O | Discontinued |
| 66 | HEETS GOLD LABEL | O | O | Discontinued |
| 67 | HEETS GOLD LABEL SELECTION | O | X | Discontinued |
| 68 | HEETS SILVER LABEL | O | O | Discontinued |
| 69 | HEETS BRONZE LABEL | O | O | Discontinued |
| 70 | HEETS BLACK GREEN SELECTION | O | O | Obtained |
| 71 | HEETS BLACK PURPLE SELECTION | O | O | Discontinued |
| 72 | HEETS SUMMER WAVE (BREEZE) | O | O | Obtained |
| 73 | HEETS TURQUOISE LABEL | O | O | Discontinued |
| 74 | HEETS YUGEN | O | O | Discontinued |
| 75 | HEETS AMMIL | O | X | Discontinued |
| 76 | HEETS SATIN LABEL | O | O | Discontinued |
| 77 | NEO NEOSTICKS SWITCH | O | O | Obtained |
| 78 | NEO NEOSTICKS BOOST | O | O | Obtained |
| 79 | NEO NEOSTICKS PURPLE BOOST | O | O | Obtained |
| 80 | NEO NEOSTICKS MAX BOOST | O | O | Obtained |
| 81 | NEO NEOSTICKS BRIGHT TOBACCO | O | O | Obtained |
| 82 | NEO NEOSTICKS DARK TOBACCO | O | O | Obtained |
| 83 | NEO NEOSTICKS FRESH | O | O | Obtained |
| 84 | NEO NEOSTICKS FRESH MIX | O | X | Discontinued |
| 85 | NEO NEOSTICKS PURPLE FRESH | O | X | Discontinued |
| 86 | NEO NEOSTICKS RUBY FRESH | O | X | Discontinued |
| 87 | NEO NEOSTICKS SMOOTH FRESH | O | X | Discontinued |
| 88 | NEO NEOSTICKS ZEST MIX | O | O | Discontinued |
| 89 | NEO NEOSTICKS ZEST COOL | O | O | Obtained |
| 90 | NEO NEOSTICKS TROPICAL COOL | X | O | Discontinued |
| 91 | NEO NEOSTICKS ICE TROPIC DOUBLE | O | O | Obtained |
| 92 | (HYPER) NEO BOOST | O | O | Low-demand |
| 93 | (HYPER) NEO PURPLE BOOST | X | O | Obtained |
| 94 | (HYPER) NEO SHINE BOOST | X | O | Low-demand |
| 95 | (HYPER) NEO DARK TOBACCO | X | O | Low-demand |
| 96 | (HYPER) NEO RUBY COOL | O | X | Discontinued |
| 97 | (HYPER) NEO FRESH | X | O | Discontinued |
| 98 | (HYPER) NEO TROPICAL COOL | O | X | Discontinued |
| 99 | (HYPER) NEO TOBACCO SWITCH | X | O | Low-demand |

**Table S3. General Characteristics of Samples (N=214)**

| **Variable** | **Total** | | **Cigarette** | | **Heated tobacco product (HTP)** | |
| --- | --- | --- | --- | --- | --- | --- |
|  | **(N=214)** | | **(n=150)** | | **(n=64)** | |
|  | **Count** | **Percentage** | **Count** | **Percentage** | **Count** | **Percentage** |
| **Manufacturer** |  |  |  |  |  |  |
| British American Tobacco (BAT) | 31 | 14.5 | 21 | 14.0 | 10 | 15.6 |
| Japan Tobacco Internaional (JTI) | 26 | 12.1 | 26 | 17.3 | 0 | 0.0 |
| Philip Morris International Korea (PMIK) | 49 | 22.9 | 28 | 18.7 | 21 | 32.8 |
| KT&G | 108 | 50.5 | 75 | 50.0 | 33 | 51.6 |
| **Price (KRW)** | **(Total) Minimum: 3,000 KRW Median: 4,500 KRW Mean: 4,558 KRW Maximum: 7,000 KRW** | | | | | |
| 3000-3900 KRW | 2 | 0.9 | 2 | 1.3 | 0 | 0.0 |
| 4000~4400 KRW | 17 | 7.9 | 17 | 11.3 | 0 | 0.0 |
| 4500-4900 KRW | 184 | 86.0 | 120 | 80.0 | 64 | 100.0 |
| 5000-7000 KRW | 11 | 5.1 | 11 | 7.3 | 0 | 0.0 |
| **Number of sticks per pack** |  |  |  |  |  |  |
| 14 | 1 | 0.5 | 1 | 0.7 | 0 | 0.0 |
| 20 | 213 | 99.5 | 149 | 99.3 | 64 | 100.0 |
| **Tar content (mg)** | **(Total) Minimum: 0.1mg Median: 1.5mg Mean: 2.7mg Maximum: 8.0mg** | | | | | |
| 0.1-0.9 mg | 7 | 3.3 | 7 | 4.7 | 0 | 0.0 |
| 1.0-1.9 mg | 72 | 33.6 | 72 | 48.0 | 0 | 0.0 |
| 2.0-2.9 mg | 3 | 1.4 | 3 | 2.0 | 0 | 0.0 |
| 3.0-3.9 mg | 25 | 11.7 | 25 | 16.7 | 0 | 0.0 |
| 4.0-4.9 mg | 5 | 2.3 | 5 | 3.3 | 0 | 0.0 |
| 5.0-5.9 mg | 15 | 7.0 | 15 | 10.0 | 0 | 0.0 |
| 6.0-6.9 mg | 20 | 9.3 | 20 | 13.3 | 0 | 0.0 |
| 7.0-7.9 mg | 0 | 0.0 | 0 | 0.0 | 0 | 0.0 |
| 8.0-8.9 mg | 3 | 1.4 | 3 | 2.0 | 0 | 0.0 |
| NA (HTPs) | 64 | 29.9 | 0 | 0.0 | 64 | 100.0 |
| **Nicotine content (mg)** | **(Total) Minimum: 0.01mg Median: 0.15mg Mean: 0.24mg Maximum: 0.70mg** | | | | | |
| 0.01-0.09 mg | 9 | 4.2 | 9 | 6.0 | 0 | 0.0 |
| 0.10-0.19 mg | 70 | 32.7 | 70 | 46.7 | 0 | 0.0 |
| 0.20-0.29 mg | 16 | 7.5 | 16 | 10.7 | 0 | 0.0 |
| 0.30-0.39 mg | 14 | 6.5 | 14 | 9.3 | 0 | 0.0 |
| 0.40-0.49 mg | 13 | 6.1 | 13 | 8.7 | 0 | 0.0 |
| 0.50-0.59 mg | 19 | 8.9 | 19 | 12.7 | 0 | 0.0 |
| 0.60-0.69 mg | 6 | 2.8 | 6 | 4.0 | 0 | 0.0 |
| 0.70-0.79 mg | 3 | 1.4 | 3 | 2.0 | 0 | 0.0 |
| NA (HTPs) | 64 | 29.9 | 0 | 0.0 | 64 | 100.0 |
| **Pack depth (mm)** | **(Total) Minimum: 40mm Median: 56mm Mean: 60mm Maximum: 79mm** | | | | | |
| 40-49 mm | 4 | 1.9 | 3 | 2.0 | 1 | 1.6 |
| 50-59 mm | 156 | 72.9 | 147 | 98.0 | 9 | 14.1 |
| 60-69 mm | 0 | 0.0 | 0 | 0.0 | 0 | 0.0 |
| 70-79 mm | 54 | 25.2 | 0 | 0.0 | 54 | 84.4 |
| **Pack width (mm)** | **(Total) Minimum: 11mm Median: 15mm Mean: 17mm Maximum: 23mm** | | | | | |
| 10-14 mm | 66 | 30.8 | 57 | 38.0 | 9 | 14.1 |
| 15-19 mm | 58 | 27.1 | 4 | 2.7 | 54 | 84.4 |
| 20-24 mm | 90 | 42.1 | 89 | 59.3 | 1 | 1.6 |
| **Pack height (mm)** | **(Total) Minimum: 47mm Median: 87mm Mean: 82mm Maximum: 103mm** | | | | | |
| 40-49 mm | 21 | 9.8 | 0 | 0.0 | 21 | 32.8 |
| 50-59 mm | 33 | 15.4 | 0 | 0.0 | 33 | 51.6 |
| 60-69 mm | 0 | 0.0 | 0 | 0.0 | 0 | 0.0 |
| 70-79 mm | 1 | 0.5 | 0 | 0.0 | 1 | 1.6 |
| 80-89 mm | 96 | 44.9 | 87 | 58.0 | 9 | 14.1 |
| 90-99 mm | 0 | 0.0 | 0 | 0.0 | 0 | 0.0 |
| 100-109 mm | 63 | 29.4 | 63 | 42.0 | 0 | 0.0 |
| **Pack shape** |  |  |  |  |  |  |
| Rectangle | 136 | 63.6 | 82 | 54.7 | 54 | 84.4 |
| Round edged rectangle | 48 | 22.4 | 45 | 30.0 | 3 | 4.7 |
| Half round edged rectangle | 30 | 14.0 | 23 | 15.3 | 7 | 10.9 |
| **Cigarette stick length (mm)** | **(Total) Minimum: 45mm Median: 83mm Mean: 79mm Maximum: 102mm** | | | | | |
| 40-49 mm | 54 | 25.2 | 0 | 0.0 | 54 | 84.4 |
| 50-59 mm | 0 | 0.0 | 0 | 0.0 | 0 | 0.0 |
| 60-69 mm | 0 | 0.0 | 0 | 0.0 | 0 | 0.0 |
| 70-79 mm | 1 | 0.5 | 0 | 0.0 | 1 | 1.6 |
| 80-89 mm | 96 | 44.9 | 87 | 58.0 | 9 | 14.1 |
| 90-99 mm | 34 | 15.9 | 34 | 22.7 | 0 | 0.0 |
| 100-109 mm | 29 | 13.6 | 29 | 19.3 | 0 | 0.00 |
| **Health warning compliance** |  |  |  |  |  |  |
| Yes | 214 | 100.0 | 150 | 100.0 | 64 | 100.0 |
| No | 0 | 0.0 | 0 | 0.0 | 0 | 0.0 |
| **Marketing text (excluding brand name)** |  |  |  |  |  |  |
| Yes | 137 | 64.0 | 103 | 68.7 | 34 | 53.1 |
| No | 77 | 36.0 | 47 | 31.3 | 30 | 46.9 |

**Figure S1. Tobacco Product Photography Guideline (Before Opening Tobacco Product Pack)**


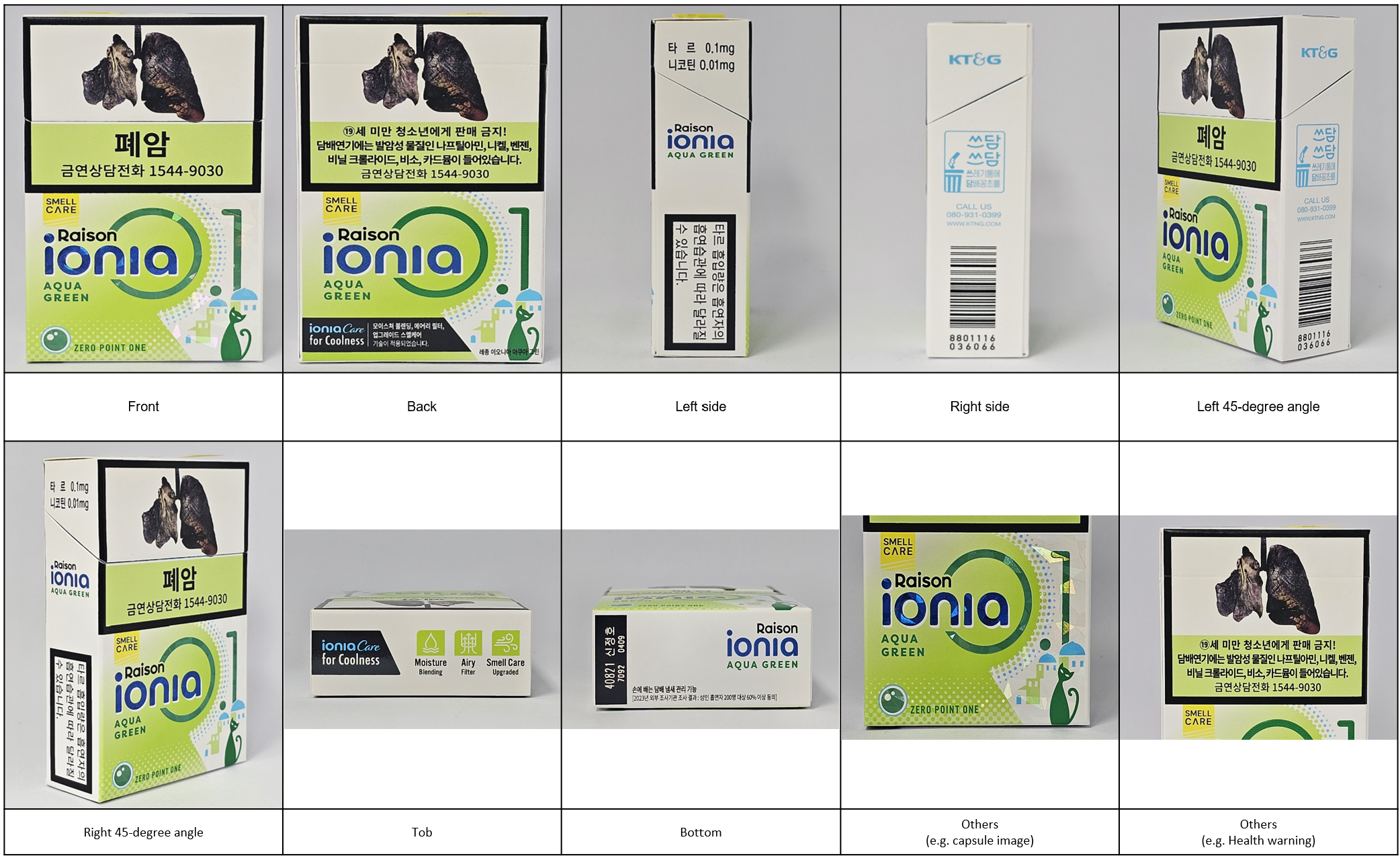


**Figure S2. Tobacco Product Photography Guideline (After Opening Tobacco Product Pack)**


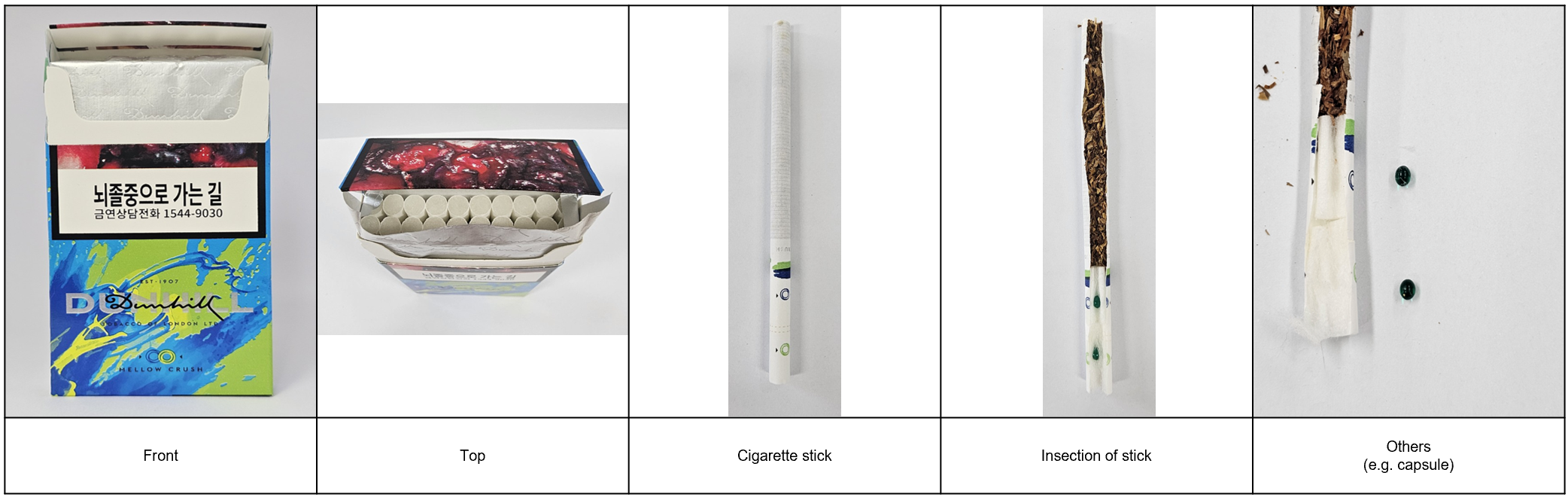


**Figure S3. Components of Flavor Cues**


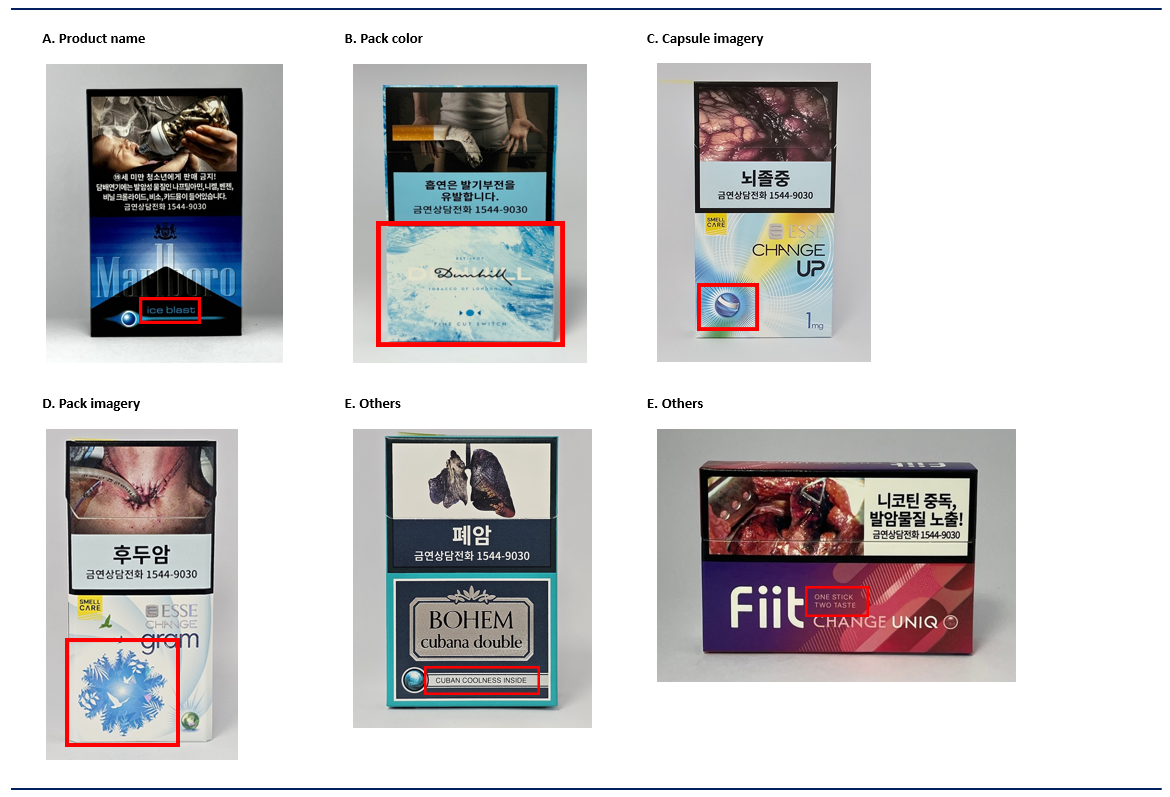


**Figure S4. Components of Tobacco Products**


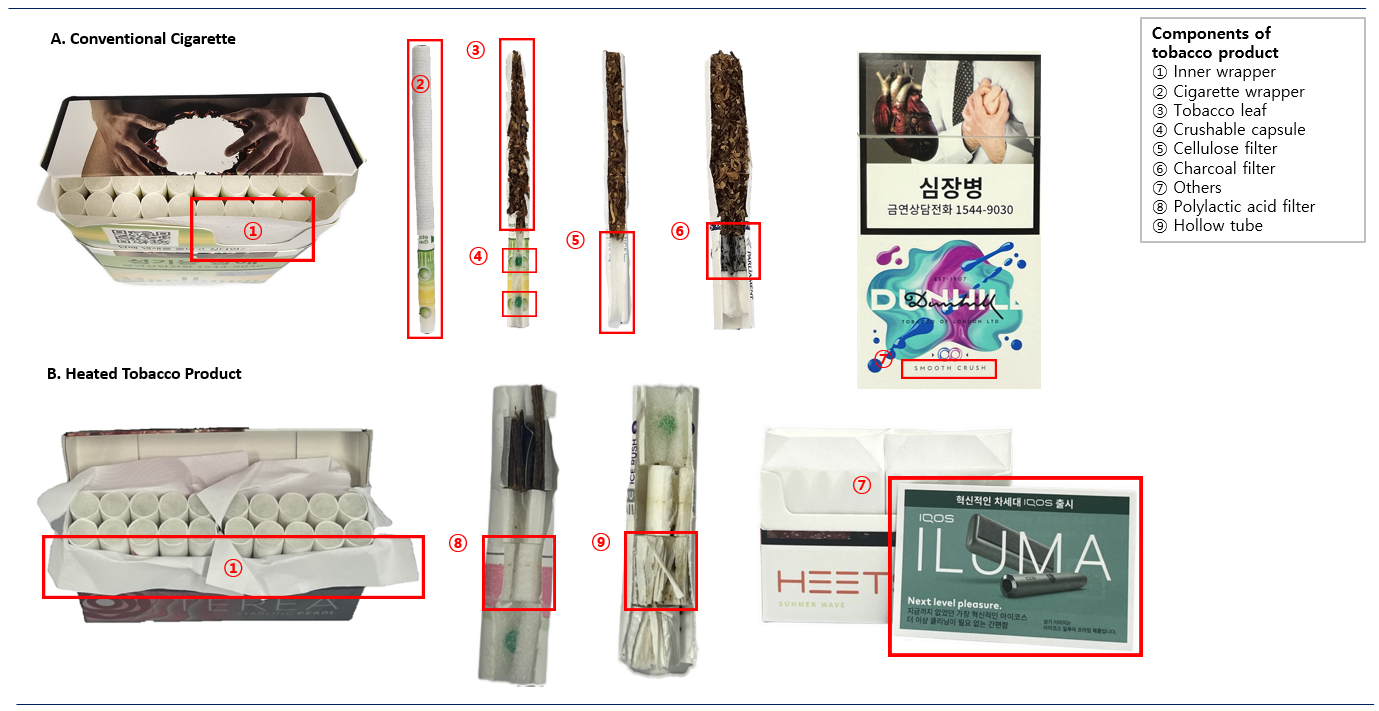


**Figure S5. Example of stick-level capsule imagery and color-group classification**


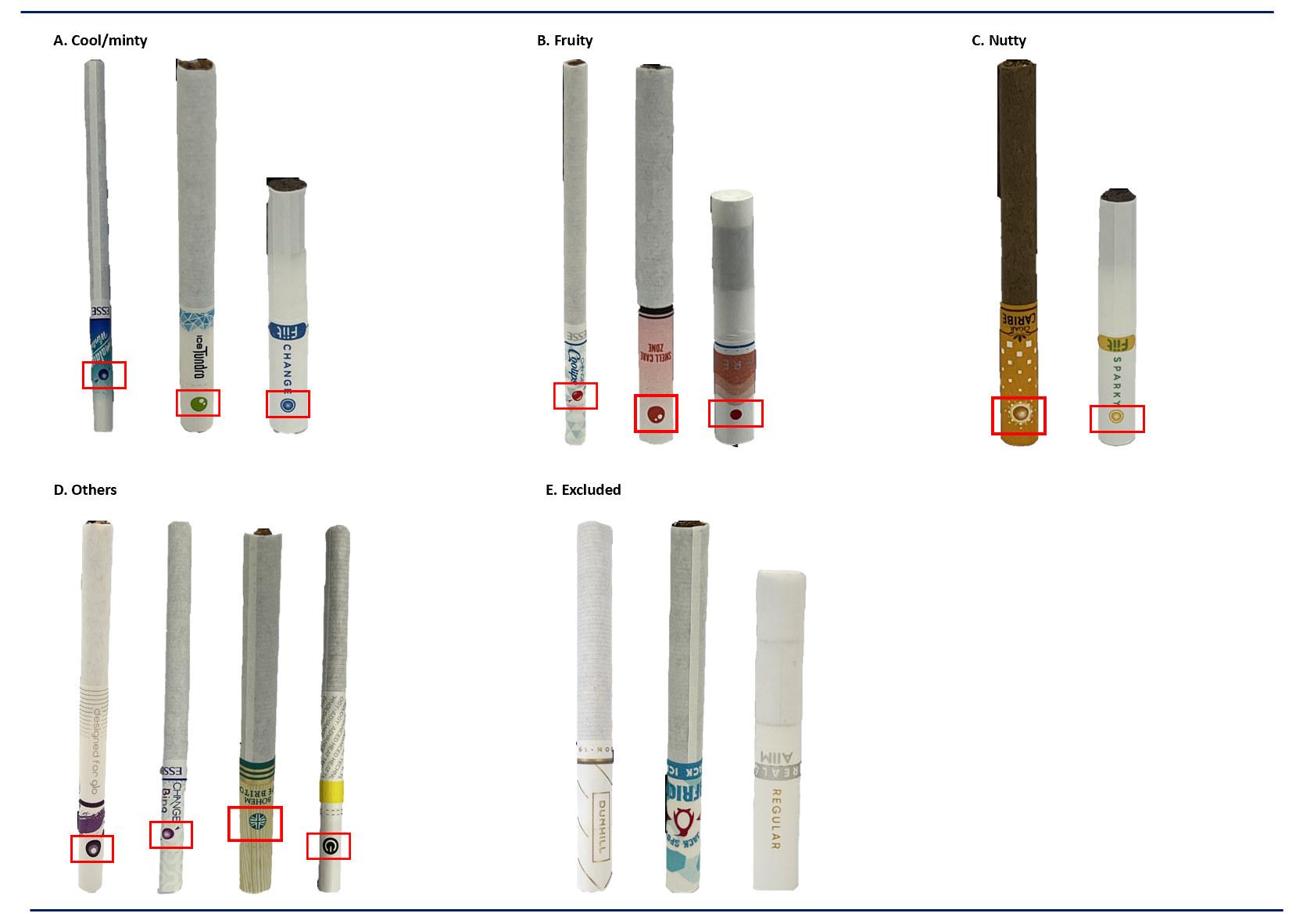


**Figure S6. Comparison of Tobacco Product Pack by Dimension**


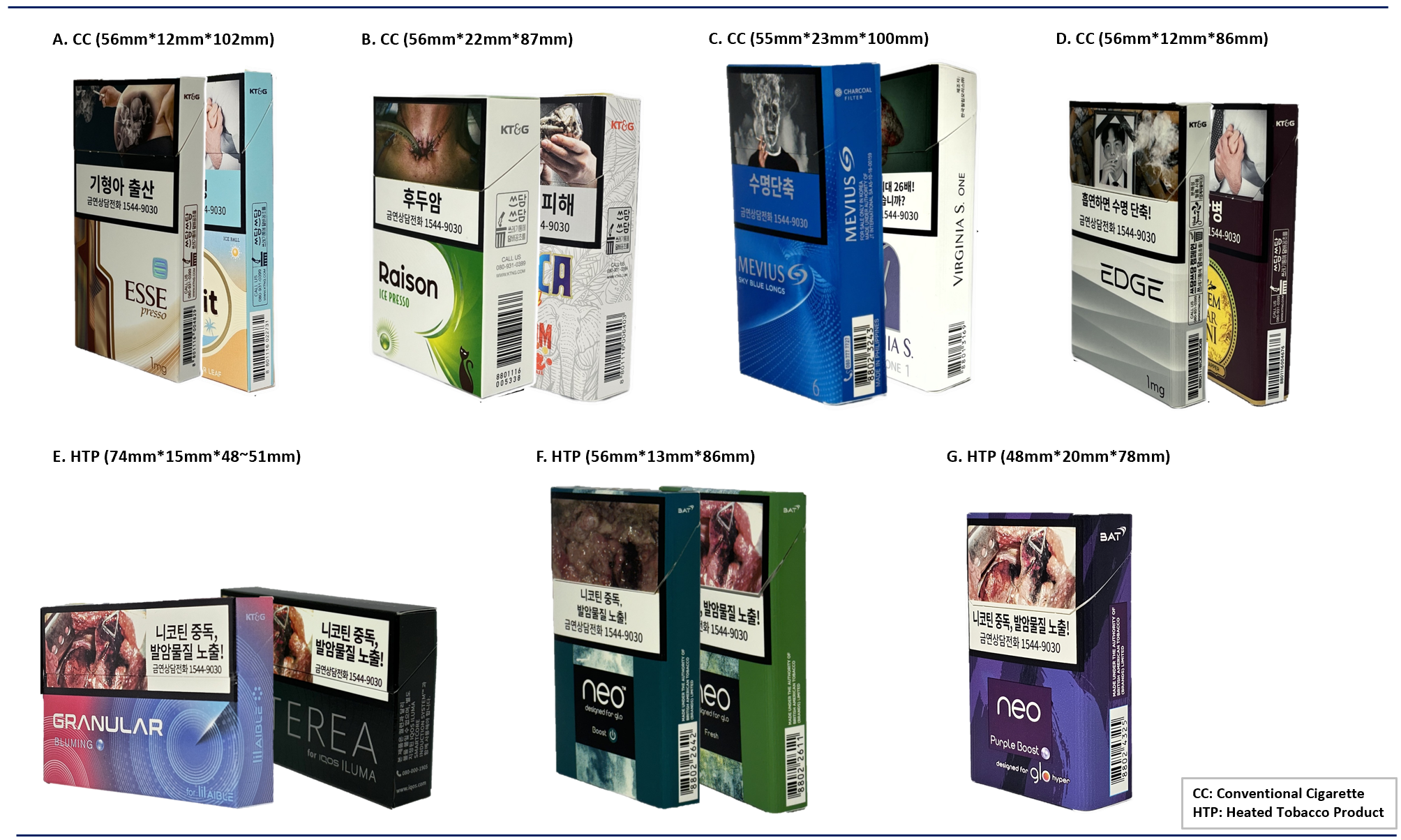

Supplement: Multimedia Appendix 1 [file publichealth-v12-e87537-s001.docx]
